# Postoperative otorhinolaryngologic complications in transnasal endoscopic surgery to access the skull base^☆^

**DOI:** 10.1016/j.bjorl.2016.04.020

**Published:** 2016-05-31

**Authors:** Ricardo Landini Lutaif Dolci, Marcel Menon Miyake, Daniela Akemi Tateno, Natalia Amaral Cançado, Carlos Augusto Correia Campos, Américo Rubens Leite dos Santos, Paulo Roberto Lazarini

**Affiliations:** a Irmandade da Santa Casa de Misericórdia de São Paulo, Departamento de Otorrinolaringologia, São Paulo, SP, Brazil; b Faculdade de Ciências Médicas da Santa Casa de São Paulo (FCMSC-SP), São Paulo, SP, Brazil; c Irmandade da Santa Casa de Misericórdia de São Paulo, Departamento de Cirurgia, Disciplina de Neurocirurgia, São Paulo, SP, Brazil

**Keywords:** Nasoseptal flap, Endonasal, Endoscopic, Skull base, Complications, Otorhinolaryngologic, Retalho nasoseptal, Endonasal, Endoscópica, Base do crânio, Complicações, Otorrinolaringológicas

## Abstract

**Introduction:**

The large increase in the number of transnasal endoscopic skull base surgeries is a consequence of greater knowledge of the anatomic region, the development of specific materials and instruments, and especially the use of the nasoseptal flap as a barrier between the sinus tract (contaminated cavity) and the subarachnoid space (sterile area), reducing the high risk of contamination.

**Objective:**

To assess the otorhinolaryngologic complications in patients undergoing endoscopic surgery of the skull base, in which a nasoseptal flap was used.

**Methods:**

This was a retrospective study that included patients who underwent endoscopic skull base surgery with creation of a nasoseptal flap, assessing for the presence of the following post-surgical complications: cerebrospinal fluid leak, meningitis, mucocele formation, nasal synechia, septal perforation (prior to posterior septectomy), internal nasal valve failure, epistaxis, and olfactory alterations.

**Results:**

The study assessed 41 patients undergoing surgery. Of these, 35 had pituitary adenomas (macro- or micro-adenomas; sellar and suprasellar extension), three had meningiomas (two tuberculum sellae and one olfactory groove), two had craniopharyngiomas, and one had an intracranial abscess. The complications were cerebrospinal fluid leak (three patients; 7.3%), meningitis (three patients; 7.3%), nasal fossa synechia (eight patients; 19.5%), internal nasal valve failure (six patients; 14.6%), and complaints of worsening of the sense of smell (16 patients; 39%). The olfactory test showed anosmia or hyposmia in ten patients (24.3%). No patient had mucocele, epistaxis, or septal perforation.

**Conclusion:**

The use of the nasoseptal flap has revolutionized endoscopic skull base surgery, making the procedures more effective and with lower morbidity compared to the traditional route. However, although mainly transient nasal morbidities were observed, in some cases, permanent hyposmia and anosmia resulted. An improvement in this technique is therefore necessary to provide a better quality of life for the patient, reducing potential complications.

## Resumo

**Introdução:**

O grande crescimento no número de cirurgias endoscópicas transnasais para a base do crânio ocorreu a partir de um maior conhecimento anatômico da região; do desenvolvimento de materiais e instrumentais específicos e, principalmente, após a utilização do retalho nasoseptal como uma barreira entre o trato sinusal (cavidade contaminada) e o espaço subaracnóideo (área estéril), com redução de grandes riscos de contaminação.

**Objetivo:**

Avaliar as complicações otorrinolaringológicas nos pacientes submetidos à cirurgia endoscópica da base do crânio, na qual foi utilizado o retalho nasoseptal.

**Método:**

Estudo retrospectivo, no qual foram avaliados os pacientes submetidos à cirurgia da base do crânio por via endoscópica com realização do retalho nasoseptal, quanto à presença no pós operatório das seguintes complicações: fístula liquórica, meningite, formação de mucocele, sinéquia nasal, perfuração septal (anterior à septectomia posterior), insuficiência de válvula nasal interna, epistaxe e alteração olfatória.

**Resultados:**

Foram avaliados 41 pacientes submetidos à cirurgia. Destes, 35 eram portadores de adenomas hipofisários (macro ou microadenomas; selares e extensão supraselar), três meningiomas (dois de tubérculo selar e um da goteira olfatória), dois craniofaringiomas e um abscesso intracraniano. As complicações observadas foram: fístula liquórica (três pacientes – 7,3%), meningite (três pacientes – 7,3%), sinéquia em fossa nasal (oito pacientes – 19,5%), insuficiência de válvula nasal interna (seis pacientes – 14,6%), e queixa de piora do olfato (16 pacientes – 39%). O teste olfatório evidenciou anosmia ou hiposmia em 10 pacientes (24,3%). Nenhum paciente apresentou mucocele, epistaxe ou perfuração septal.

**Conclusão:**

O uso do retalho nasoseptal proporcionou uma revolução na cirurgia da base do crânio por via endoscópica tornando os procedimentos mais eficazes e com baixa morbidade, comparando com a via tradicional. Porém, passou a ocasionar morbidades nasais principalmente transitórias, mas em alguns casos permanentes, como hiposmia e anosmia. Assim, torna-se necessário um aperfeiçoamento desta técnica para proporcionar uma melhoria na qualidade de vida do paciente e diminuir possíveis complicações.

## Introduction

The studies by Jho and Carrau in the 1990s demonstrated that the endoscopic sinonasal route allowed for direct access to various skull base disorders, including tumors of the pituitary gland. This was a very significant step in the development of endoscopic skull base surgery. In this new field, several studies have shown the numerous possibilities of treatment of diseases in that region; together with recent advances in anatomical knowledge and surgical techniques, they have fostered the expansion of transnasal endoscopic skull base surgery. As a result of improved optics, hemostatic equipment, specific clamps, microdebriders, diagnostic tests, and the use of neuronavigation, there was an improvement in technical efficiency, reducing morbidity, mortality and surgical time.^1^, ^2^, ^3^, ^4^, ^5^, ^6^

In the early stages of the endonasal endoscopic approach to the skull base, the main criticism was the high rates of cerebrospinal fluid (CSF) leakage, resulting in a very high mortality compared to the traditional route. The validation of this technique occurred after the development of the nasoseptal flap, which helped create an effective barrier between the sinonasal tract and the subarachnoid space, avoiding the contact of an infected cavity with a sterile area at great risk of infection.^7^, ^8^

There are several studies in the literature on the closure of skull base defects using nasoseptal flap in a wide variety of diseases and skull base locations^7^, ^8^, ^9^, ^10^; few studies have demonstrated the potential morbidities and how to avoid them. Thus, this study aimed to evaluate possible sinonasal complications in patients undergoing skull base surgery by transnasal endoscopy.

## Methods

Data collected from medical records of patients operated by the skull base team of this institution, composed of otorhinolaryngologists and neurosurgeons, were retrospectively assessed. All patients included in the study underwent endonasal endoscopic surgical procedures, with transsphenoidal, transplanum, or transcribriform approach to the base of the skull and use of nasoseptal flap, from November 2012 to December 2014, and with a minimum of 100 days of follow-up after surgery. Patients who did not undergo the endonasal access exclusively to the skull base, as well as those in which the nasoseptal flap was not used, were excluded. Patients were evaluated for demographic data and for the presence of CSF leak, meningitis, mucocele formation, olfactory changes, nasal synechiae, epistaxis, and septal perforation in the postoperative period. The data collected referred to the last patient visit and were based on reports of complaints from patients, otorhinolaryngology physical examination, nasal endoscopy, and imaging tests (computed tomography [CT] and magnetic resonance imaging [MRI]) for the diagnosis of mucoceles. Patients with olfactory alterations complaints were submitted to the smell test (Connecticut Chemosensory Clinical Research Center Test [CCCRC]). The study was approved by the Ethics Committee (CAAE – 34487814.8.0000.5479).

## Results

Forty-five patients were operated from November 2012 to December 2014, of whom 41 met the inclusion criteria, undergoing endoscopic skull base surgery: 28 females and 13 males. The mean age was 46 years (15–79 years) and mean follow-up time was 12.2 months (3.5–30 months). Four patients were excluded due to non-use of nasoseptal flap (reoperation) or loss to follow-up.

Thirty-five patients had pituitary adenomas, three had meningiomas (two tuberculum sellae and one olfactory groove), two had craniopharyngiomas, and one had a brain abscess (Table 1).

**Table 1:** Cases of endonasal endoscopic surgery of the skull base.

Pathology	Number (%)
**Adenomas**	35 (85.3)
Multi-producer	7 (17)
Non-producer	10 (24.4)
ACTH	11 (26.8)
GH	6 (14.6)
FSH	1 (2.4)
**Meningioma**	3 (7.3)
Tuberculum sellae	2 (4.8)
Olfactory groove	1 (2.4)
**Craniopharyngioma**	2 (4.8)
**Brain abscess**	1 (2.4)

Forty-one cases of endoscopic endonasal surgery of the skull base were performed with nasoseptal flap creation.

**Figure 1:**
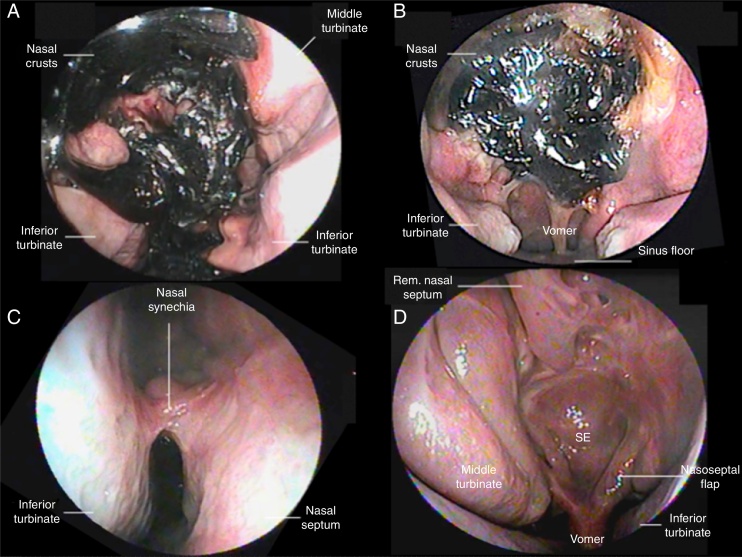
(A) and (B) Nasofibroscopy at two weeks postoperative in patients who underwent transsphenoidal surgery for pituitary adenoma, a wide number of crusts throughout the nasal cavity can be observed. It is not possible to observe the sphenoid sinus and sellar regions. No manipulation is performed in the surgical area due to the risk of manipulation of nasoseptal flap, which can result in a cerebrospinal fluid leak. (C) The right nasal fossa synechia can be observed between the inferior turbinate and the nasal septum, in the postoperative period of three months; (D) six months postoperative of pituitary adenoma, in which various anatomical structures can be identified, including the right middle turbinate as the nasoseptal flap was made to the left (the beginning of flap within the sphenoid sinus floor can be observed), with removal of the ipsilateral middle turbinate as the patient had a significant septal deviation to the right. It is also possible to observe the sphenoid region wide open and completely covered by the flap; it is possible to identify the upper portion of the nasal septum, as the nasal septum was removed (preserving the area K, Keystone, and 1 cm from the nasal septum superiorly).

For educational purposes, complications were divided according to their location (as previously described by Soudry et al.^11^), the integrity of the nasoseptal flap, the receiving area (skull base), and the donor area (septum and nasal mucosa; Table 2; Fig. 1).

**Table 2:** Complications from endonasal endoscopic surgery of the skull base.

	Number (%)
**Complications of the donor area**	
Synechia	8 (19.5)
No clinical repercussion	4 (9.7)
With clinical repercussion	4 (9.7)
Nasal valve insufficiency	7 (17)
Associated with synechia	4 (9.7)
Associated with septal deviation	2 (4.8)
Nasal wing collapse prior to surgery	1 (2.4)
Complaint of olfactory alteration	16 (39)
Anosmia	4 (9.7)
Severe hyposmia	3 (7.3)
Moderate hyposmia	2 (4.8)
Mild hyposmia	1 (2.4)
Normosmia	4 (9.7)
Did not perform the test	2 (4.8)
Mucocele	0 (0)
Septal Perforation	0 (0)

**Complications of the receptor area**	
Cerebrospinal fluid leaks	3 (7.3)
Meningitis	3 (7.3)
**Flap integrity**	NC

Otorhinolaryngologic complications of endoscopic surgery of the skull base were divided according to the location of morbidity: donor area refers to the mucosa and the nasal septum and represents the main complaints of patients after surgery, including synechiae, internal nasal valve alterations, mucocele, septal perforation, and olfactory alterations. Receptor area refers to the location of the defect in the skull base and the barrier to be created to prevent communication between sterile and contaminated areas, in which the most severe complications are observed, such as cerebrospinal fluid leak and meningitis. The integrity of the nasoseptal flap was observed in all patients in the present study. NC, no complications.

### Integrity of the flap

There were no complications directly related to the flap. In all patients, the flap had good postoperative viability (no necrosis). There were no pedicle transections during its construction in surgery.

### Receiving area

Three cases (7.3%) of CSF leaks were observed, two in patients undergoing surgery for adrenocorticotropic hormone (ACTH)-producing pituitary adenomas (Cushing's syndrome) and one for treatment of olfactory groove meningioma. A craniotomy was performed for the treatment of CSF leak in both patients with pituitary adenoma. In the patient with olfactory groove meningioma, a pericranial flap was made. After these procedures, the three patients showed improvement of CSF leak. These same three patients also had meningitis, and were treated with intravenous antibiotics. All showed satisfactory progress after treatment with antibiotics, without sequelae.

### Donor area

Alterations in the nasal fossa (synechiae, valve failure, or both) were observed in 11 (26.8%) patients, who were asked about the perception of nasal obstruction in comparison with the preoperative period: five reported worsening in breathing, five reported no difference, and one reported improved breathing. This patient had had a severe nasal septum deviation prior to surgery and underwent a septoplasty at the same operation period. Regarding the changes of the nasal cavity, eight (19.5%) patients had synechia and six (14.6%) patients developed changes in the internal nasal valve; three patients had both. Thus, among the 41 patients studied, five reported worsening in breathing (12.1%). No patients presented septal perforation, epistaxis, or mucocele.

Regarding olfactory function, 16 patients (39%) reported post-operative complaints (after over 100 days of follow-up) and were subjected to the CCCRC (Table 3, Table 4). Of these, ten (24.3%) had some degree of hyposmia or anosmia (two also showed deviation of the septum or synechia), four (9.7%) had normosmia, and two (4.8%) did not undergo the test (one patient due to loss to follow-up and the other due to cognitive impairment that prevented the application of the CCCRC). About the ten patients that had olfaction alteration, the access route were: transsphenoidal in eight patients (four multi-producer adenomas, one GH, one ACTH, and one non-producer, as well as one craniopharyngioma), and the transcribriform approach was used in two cases (one brain abscess and one olfactory groove meningioma). The patient who underwent surgery for brain abscess showed moderate hyposmia; the patient who underwent the procedure for treatment of olfactory groove meningioma presented anosmia.

**Table 3:** Complications from endonasal endoscopic surgery of the skull base, per disease.

	Olfactory alterations	Cerebrospinal fluid leak	Meningitis	Nasal synechia	INVF	P/M/E
Adenoma	7	2	2	7	5	
Craniopharyngioma	1					
Tuberculum sellae meningioma						
Olfactory groove meningioma	1	1	1			
Intracranial abscess	1			1	1	

Total	10	3	3	8	6	0

When complications were divided by surgical access, the following were observed: in three cases (7.3%), cerebrospinal fluid leak and meningitis; in the two abovementioned complications, two patients underwent surgery for adrenocorticotropic hormone (ACTH)-producer pituitary adenomas (Cushing's syndrome) and one for treatment of olfactory groove meningioma. Among the changes of the nasal cavity: in eight (19.5%) patients, synechia was observed, seven in patients undergoing surgery for pituitary adenoma, and one for intracranial abscess. Six (14.6%) patients developed changes in the internal nasal valve, five patients undergoing surgery for pituitary adenoma, and one for intracranial abscess (three patients showed synechia and alteration of the internal nasal valve). The most common complication in this study was a change in olfaction, observed in ten patients, and documented by the olfactory test *Connecticut Chemosensory Clinical Research Center Test* [CCCRC]. Of these ten patients, seven patients underwent surgery for pituitary adenoma, one for a craniopharyngioma, one because of an intracranial abscess, and one for an olfactory groove meningioma. Four patients who raised the complaint postoperatively were examined and were found normosmic. Two patients with the complaint did not undergo the examination; one was lost to follow-up and another due to cognitive impairment. No patients developed septal perforation, epistaxis, or mucocele. INVF, internal nasal valve failure; P/M/E, septal perforation/mucocele/epistaxis.

**Table 4:** Result of the Connecticut Chemosensory Clinical Research Center olfactory test after surgical approach.

Patient	BT-L	BT-R	SI-L	SI-R	CS-L	CS-R	FS	Classification
1	3	2	7	7	5	4.5	4.75	Moderate hyposmia
2	10	5	6	6	8	5.5	6.75	Normosmia
3	0	0	5	7	2.5	3.5	3	Severe hyposmia
4	8	10	7	7	7.5	8.5	8	Normosmia
5	0	O	7	7	3.5	3.5	3.5	Severe hyposmia
6	8	0	5	4	6.5	2	4.25	Moderate hyposmia
7	0	0	4	6	2	3	2.5	Severe hyposmia
8	0	0	0	0	0	0	0	Anosmia
9	0	0	0	0	0	0	0	Anosmia
10	4	5	6	6	5	5.5	5.25	Mild hyposmia
11	10	10	7	7	8.5	8.5	8.5	Normosmia
12	NP	NP	NP	NP	NP	NP	NP	
13	1	10	7	7	4	8.5	6.25	Normosmia
14	NP	NP	NP	NP	NP	NP	NP	
15	O	O	O	O	O	O	O	Anosmia
16	O	O	O	O	O	O	O	Anosmia

This test consists of two parts, consisting of the olfactory threshold of research and smell identification; the olfactory classification is performed by analyzing: (1) the combined score between the threshold test (butanol) and smell identification, which corresponds to the arithmetic mean of both scores. Thereafter, a combined score was obtained for each nasal cavity separately. (2) The Combined Score Index, which is the arithmetic mean of the combined scores of each nasal cavity. Thus, according to the combined score of the indexes obtained, the following values were considered for the classification of the olfactory status of each patient: 6.0–8.5: normosmia; 5.0–5.75: mild hyposmia; 4.0–4.75: moderate hyposmia; 2.0–3.75: severe hyposmia; and 0–1.75: anosmia. R, right; L, left; CS, combined score; FS, final score; SI, smell identification; BT, butanol threshold; NP, not performed.

## Discussion

The surgical approach to the skull base via the endonasal endoscopic access is already considered the route of choice for the treatment of various disorders, particularly pituitary adenomas. However, the need to manipulate the nasal cavity and the paranasal sinuses, as well as the preparation of the flap nasoseptal, may result in sinonasal complications. The establishment of a multidisciplinary team including neurosurgeons and otorhinolaryngologists has proven to be essential in the surgical and postoperative management of these patients, and has been adopted in the main centers worldwide.^12^ This study is one of first in Brazil to report the otorhinolaryngologic complications of the endonasal endoscopic approach to the skull base and the experience of a tertiary public hospital.

In the present study, no complications related to flap integrity were observed, as flap construction was standardized to the right, with variation when a nasal septum deviation hindered its construction to the right; this reduced the chances of complications in making the flap. Another factor that helped flap viability was the maintenance of a pedicle by performing a lower and upper incision in the choanal arch, with a distance of at least 1–1.5 cm, as the lower incision is made in the lower arch of the choana and the top incision just below the sphenoid ostium.

Before the advent of the nasoseptal flap, complications arising from the receiving area were a limiting factor due to a high CSF leak rate, which ranged from 40% to 50% in the endoscopic approach. These rates were considered unacceptable due to high morbidity and mortality, and the main criticism was that there was a traditional and renowned access route with lower complication rates.^8^, ^11^, ^13^, ^14^ Currently, the percentage of postoperative CSF leak is approximately 5%.^7^, ^15^ In the present study, a rate of 7.3% was observed; however, this rate included cases of expanded endoscopic approach (three meningiomas [two tuberculum sellae and one olfactory groove meningioma], two craniopharyngiomas, and one brain abscess), and a CSF leak was observed in the olfactory groove meningioma. If only the pituitary surgeries are considered, a CSF leak rate of 5.7% was observed. When the expanded endoscopic approach to the skull base is performed, CSF leak rates are higher, as there is high CSF flow, leading to a greater propensity of leaks. Rates vary from 10% to 16.1%.^9^, ^16^, ^17^ In this study, analyzing only the expanded access, the CSF leak rate was 16.6%.

The highest rates of complications arise from the donor area. Olfactory complications have been previously studied; some studies have shown a direct relationship between the construction of nasoseptal flap and possible worsening of symptoms, with rates ranging from 8% to 46%.^18^, ^19^, ^20^, ^21^, ^22^, ^23^ The present study diagnosed anosmia or some degree of hyposmia (mild to severe) in ten patients (24.4%) through CCCRC. If both patients with olfactory complaint who were not tested were diagnosed, this ratio would reach 29.2%. Unlike some studies that included only patients who were operated on using the transsphenoidal access, the present sample included two patients in whom the transcribriform access was used, which has a worse olfactory prognosis.

Several factors are related to olfactory alterations. In the first month, the presence of crusts in the nasal cavity resulting from surgical procedures play an important role and can persist for 30–100 days.^20^, ^24^, ^25^ However, these crusts occur transiently; with the correct cleaning of the nasal cavity, there can be a significant improvement in the sense of smell. In addition, other factors that can minimize olfactory complaints are the preservation of 2 cm from the upper region of the nasal septum mucosa during the making of the flap, as this region is rich in olfactory neuroepithelium, and the preservation of the middle and upper turbinates, structures with the presence of olfactory fimbriae.

Another complication arising from the donor area, and one of the most common, is nasal synechia, which occurs between the septum and structures of the lateral nasal wall, that can cause worsening of nasal flow. In the present study, eight cases of postoperative synechiae (19.5%) were identified, a percentage similar to previous reports in the literature, which ranged from 9% to 20%.^21^, ^24^ In the present authors’ experience, the main causes of synechiae are the lack of proper nasal lavage with saline in the postoperative period and failure to place the nasal splint, exposing areas of bloody septal mucosa.

Internal nasal valve failure is also a common complication, which was observed in six (14%) of the cases studied, four due to adhesions and two due to residual septal deviations. Another possible cause of internal nasal valve failure is called ‘alar sill burn’ due to synechia of the lower lateral cartilage and the nasal septum. This is reported in 5% of patients following creation of the upper incision of nasoseptal flap with electrocautery in the anterior portion of the nasal septum, and can be avoided by placing the nasal splint.^24^ In the present study, no cases were observed.

Although rates of septal perforation of up to 14% have been described,^11^ no patient in the present study had this complication. The creation of the reverse flap, covering the remaining cartilaginous septum from where nasoseptal flap was removed, is routinely performed in this service, and the authors believe it to be very important for proper healing.^26^ In the present study, no cases of mucocele were observed; this has been reported in previous studies between 0% and 50%,^27^, ^28^, ^29^, ^30^, ^31^ being more frequent in children due to incomplete formation of the frontal sinus in cases of access to the anterior skull base.^31^, ^32^ The authors believe that the following steps are critical to minimize the development of mucocele, especially in the sphenoid: complete removal of the mucosa and all septae present in the sphenoid sinus, making it easier for the flap to adhere; mucosal denudation in the area around the defect of the skull base; wide sphenoidectomy, thus reducing the risk of blockage of airflow; and avoiding overlap between flaps in cases of double flap.^29^, ^33^ In the present study, no cases of epistaxis were observed that required medical or surgical intervention, as all patients were maintained postoperatively with a Foley catheter and nasal splint for three to seven days, resulting in less chance of bleeding.

The study limitations relate to the fact that it was performed retrospectively. There is a need for further studies associating endoscopic skull base surgery with possible olfactory alterations. The smell test was performed only postoperatively and in patients with complaints.

Currently, this technique is well established as the best way to access the skull base. Studies such as the present indicate the need for further technical improvement to avoid any complications and thus provide healing and better well-being for patients.

## Conclusion

The transnasal endoscopic technique is no longer a novelty and has become a reality, especially after the advent of nasoseptal flap. However, its use leads to nasal alterations that are usually transitory, but sometimes permanent. Among them are the olfactory alterations, which cause a decrease in the patient's quality of life. Based on the data presented in this study, it is important to improve the surgical technique so that the patient can be benefited by avoiding postoperative complaints and complications.

## Conflicts of interest

The authors declare no conflicts of interest.
